# Comprehensive Analysis and Risk Identification of Pulmonary Cryptococcosis in Non-HIV Patients

**DOI:** 10.3390/jof7080657

**Published:** 2021-08-13

**Authors:** Chun Lin, Tsung-Ying Yang, Ming-Cheng Chan, Kuo-Hsuan Hsu, Yen-Hsiang Huang, Jeng-Sen Tseng

**Affiliations:** 1 Division of Chest Medicine, Department of Internal Medicine, Taichung Veterans General Hospital, Taichung 40705, Taiwan; furioso@vghtc.gov.tw (C.L.); jonyin@gmail.com (T.-Y.Y.); waynehuang0622@gmail.com (Y.-H.H.); 2 Department of Life Sciences, National Chung Hsing University, Taichung 40227, Taiwan; 3 Division of Critical Care and Respiratory Therapy, Department of Internal Medicine, Taichung Veterans General Hospital, Taichung 40705, Taiwan; drchan@vghtc.gov.tw (M.-C.C.); khhsu@vghtc.gov.tw (K.-H.H.); 4 The College of Science, Tunghai University, Taichung 40704, Taiwan; 5 Institute of Biomedical Sciences, National Chung Hsing University, Taichung 40227, Taiwan; 6 School of Medicine, National Yang Ming Chiao Tung University, Taipei 11221, Taiwan

**Keywords:** pulmonary cryptococcosis, non-human immunodeficiency virus (HIV), comorbidity, cryptococcal antigen (CrAg), *Cryptococcus* spp.

## Abstract

Pulmonary cryptococcosis in the non-human immunodeficiency virus-infected population is uncommon. We aimed to explore the relevance between clinical presentations, radiological findings, and comorbidities and identify the outcome predictors. A total of 321 patients at Taichung Veterans General Hospital between 2005 and 2019 were included; of them, 204 (63.6%) had at least one comorbidity, while 67 (20.9%) had two or more. The most common comorbidities were diabetes mellitus (27.4%), malignant solid tumor (19.6%), autoimmune disease (15.6%), and chronic kidney disease (8.4%). Patients experiencing comorbidity, particularly those with multiple comorbidities, had a higher multilobar and extrapulmonary involvement, which could explain these patients being more symptomatic. In the overall population, extrapulmonary involvement independently predicted disease recurrence and death. Amongst patients with isolated pulmonary cryptococcosis, age, cryptococcal antigen (CrAg) titer in blood, and comorbidities not only predicted the extent of disease, but also its outcome. Of note, patients simultaneously with age ≥ 65 years, CrAg test ≥ 1:128, and multiple comorbidities had the lowest disease control of antifungal treatment (76.9%) and the highest rate of disease recurrence or death from any cause (40.0%). In conclusion, approximately two-thirds of patients had at least one underlying comorbidity. In addition to extrapulmonary involvement, old age, high CrAg titer in blood, and multiple comorbidities could act as risk factors for predicting the extent of disease and outcome.

## 1. Introduction

Cryptococcosis is an invasive fungal infection with worldwide distribution caused by *Cryptococcus* spp., particularly *Cryptococcus neoformans* and *Cryptococcus gattii* [1]. According to the ARTEMIS DISK global antifungal surveillance study [2], *Cryptococcus* is the most common genera of noncandidal yeast. In most circumstances, the infection begins in the lungs and then may spread to other organs, particularly the central nervous system (CNS). Although the respiratory system is the main entrance point for *Cryptococcus* to enter the human body, fungemia and meningitis are far more common as the presenting syndrome of cryptococcosis in both human immunodeficiency virus (HIV)- and non-HIV-infected persons [3]. Owing to the lack of large prospective studies, the optimal therapy and prognostic factors regarding pulmonary cryptococcosis have not been clearly elucidated [4,5].

Cryptococcosis mainly occurs in immunocompromised hosts. HIV infection is the most common risk factor [6]. Several non-HIV risk factors have been reported, including malignancy, organ transplantation, diabetes mellitus, and disorders which require immunosuppressant therapy [5,7,8]. Actually, cryptococcosis, particularly isolated pulmonary infection, could occur in patients without any identifiable immunocompromised situation. Since most of the data came from retrospective studies with low patient numbers, the results were not consistent; therefore, how underlying comorbidities influence clinical presentation and the outcome of treatment remains unclear.

Patients with pulmonary cryptococcosis, particularly those without HIV infection and a lack of extrapulmonary involvement, usually have minor clinical symptoms, with some of them actually being asymptomatic [9,10,11,12]. Cough, shortness of breath, fever, and chest pain are the most common symptoms of *Cryptococcus* lung disease [8,9,10]. However, limited studies have addressed the relationship between patients’ clinical presentations, radiological findings, and the extent of disease.

We conducted a large-scale study to comprehensively analyze the clinical presentations, underlying comorbidities, diagnostic methods, treatment patterns, and outcome of pulmonary cryptococcosis in non-HIV patients. We also explored the risk factors which correlate with the extent of disease, while also identifying subjects who had a poor prognosis.

## 2. Materials and Methods

### 2.1. Patients

We included patients with pulmonary cryptococcosis who had been diagnosed at Taichung Veterans General Hospital between 2005 and 2019. To be eligible for participation, patients were required to have confirmed cryptococcosis, which was determined by histopathology, serology, or microbiological cultures, as well as identifiable lung lesions on chest images related to *Cryptococcus* infection. Subjects with extrapulmonary involvement were allowed. Patients were excluded if they had only extrapulmonary cryptococcosis or were HIV infected with or without acquired immunodeficiency syndrome. This study was conducted ethically in accordance with the World Medical Association Declaration of Helsinki and was approved by the Institutional Review Board of Taichung Veterans General Hospital (IRB No. CE20109B).

### 2.2. Data Records and Response Evaluation

Clinical data for analysis included age, gender, smoking status, comorbidities, clinical symptoms at diagnosis, extent of disease, findings on chest computed tomography, diagnostic methods used, yield rate of each method, patterns of treatment, and outcome of pulmonary cryptococcosis. All chest images were reviewed by two chest physicians. Analyses regarding radiological presentations were only conducted in patients with chest computed tomography. The cryptococcal antigen (CrAg) test in our hospital was performed by Meridian Cryptococcal Antigen Latex Agglutination System (CALAS®).

The response to antifungal treatment was evaluated by the change in lung lesions seen on the chest images, with the response criteria modified from the Mycoses Study Group and European Organization for Research and Treatment of Cancer Consensus Criteria [13]. Of them, complete response was defined as survival at the end of treatment and resolution of all radiological lesion(s). Partial response was defined as survival at the end of treatment and a minimum 25% reduction in the diameter of radiological lesion(s). Stable disease was defined as survival at the end of treatment and a 0% to 25% reduction in the diameter of the radiological lesion(s). Disease control means the sum of objective response, and stable disease. Disease progression was defined as new sites of disease or radiological worsening of pre-existing lesion(s) or death from cryptococcosis before the end of treatment.

Other outcome parameters included disease recurrence, death from cryptococcosis, and death from any cause.

### 2.3. Statistical Methods

Univariate analyses of patients’ characteristics, clinical and radiological presentations, diagnostic results, and outcome of treatment were performed by Fisher’s exact test. The Kaplan–Meier method was used to estimate the median duration of treatment and follow-up. The receiver-operating characteristic (ROC) curve was implemented for evaluating the CrAg test of blood in predicting the extent of disease and outcome. The logistic regression model was used to estimate the odds of extent of disease, disease control rate, recurrence, and death, as well as to conduct multivariate analysis. All statistical tests were carried out using SPSS 15.0 (SPSS Inc., Chicago, IL, USA). Two-tailed tests and *p* values < 0.05 for significance were implemented.

## 3. Results

### 3.1. Patients’ Characteristics

A total of 321 patients were included, with their characteristics summarized in Table 1. The median age was 59 years, 138 patients (43.0%) were female, and 234 (72.9%) were non-smokers.

With regard to physical condition, a total of 204 patients (63.6%) had at least one comorbidity. Of them, 137 patients had one comorbidity, while 67 had two or more. The most common comorbidities were diabetes mellitus (27.4%), malignant solid tumor (19.6%), autoimmune disease (15.6%), and chronic kidney disease (8.4%). The most common malignancies were lung cancer, colorectal cancer, and breast cancer. The most common autoimmune diseases were rheumatoid arthritis, systemic lupus erythematosus, and Sjogren’s syndrome. Chronic kidney disease denoted patients who were at stage 3 or more (*n* = 27). Chronic lung disease (*n* = 15) recorded patients with chronic obstructive pulmonary disease or other structural lung disease who had a documented decline in lung function and required treatment. Chronic liver disease (*n* = 10) recorded patients who were at cirrhosis status but did not include subjects who were only carriers of viral hepatitis. Cardiovascular disease (*n* = 8) recorded patients with coronary artery disease or other structural heart disease with documented heart failure symptoms or signs.

### 3.2. Clinical and Radiological Presentations

The results of the clinical and radiological presentations are summarized in Table 2. With regard to the clinical presentations, 111 patients (34.6%) and 210 patients (65.4%) were asymptomatic and symptomatic, respectively. Cough (40.2%) was the most common symptom, followed by fever (13.1%), shortness of breath (7.2%), and chest pain (6.5%).

Overall, 299 patients (93.1%) had isolated pulmonary cryptococcosis. Twenty-two patients (6.9%) had extrapulmonary involvement, with CNS being the most common infective site (*n* = 18). Other diagnoses included fungemia (*n* = 8), neck lymph node (*n* = 2), paraspinal abscess (*n* = 1), and urinary tract infection (*n* = 1).

Regarding radiological presentations, we excluded 16 patients who had no computed tomography at diagnosis. Amongst the remaining cases, 185 (60.7%) and 120 patients (39.3%) had single lobe and multiple lobe involvement, respectively. The most common findings were multiple nodules or masses with or without cavitation (*n* = 161, 52.8%), consolidation (*n* = 52, 17.0%), and a solitary nodule or mass with or without cavitation (*n* = 48, 15.7%) (Supplementary Figure S1). There were patients presented with mixed patterns, the most common of which was consolidation mixed with nodule(s)/mass(es) (*n* = 29). A total of 37 patients had cavitation. Otherwise, patients presenting with pure ground glass opacity, pleural effusion, and lymphadenopathy were rare.

### 3.3. The Pattern of Diagnostic Methods and Their Yield Rates

The results of diagnostic patterns are summarized in Table 3. A total of 303 patients (94.4%) had a baseline blood CrAg test, with 250 (82.5%) being positive (median 1:32 (range 1:2 to 1:8192)). There were 110 patients (34.3%) undergoing lumbar puncture for cerebrospinal fluid (CSF) sampling, with 18 patients of them (16.4%) showing positive results. All of them also had positive results in the blood.

Microbiological cultures were obtained in 154 patients (48.0%). The most common were CSF (*n* = 110), tissue biopsy (*n* = 62), and bronchoalveolar lavage (*n* = 21). Thirteen of the 110 CSF cultures (11.8%) yielded positive for *Cryptococcus*, with all of them positive for CSF CrAg test. A total of 47 *Cryptococcus* spp. were identified, including 46 *Cryptococcus neoformans* and 1 *Cryptococcus gattii*.

A total of 135 patients (42.1%) underwent a biopsy procedure. The most common were surgical biopsy (*n* = 64) and computed-tomography-guided biopsy (*n* = 24); the yield rates of cryptococcosis were 100.0% and 85.7%, respectively.

### 3.4. The Pattern of Treatment and Outcomes

The results of the treatment patterns and outcomes are summarized in Table 4. After diagnosis, 282 patients (87.9%) underwent antifungal treatment; of them, 251 (89.0%) received fluconazole and 31 (11.0%) received an amphotericin-B-containing regimen. There were 34 patients (10.6%) who presented with limited numbers of lung lesions who underwent surgical resection only. Five patients did not receive any treatment. Except for one patient diagnosed with oral cancer and chronic obstructive pulmonary disease who declined treatment, and another who was lost during follow-up, the remaining three patients had neither a comorbidity nor any extrapulmonary involvement. At last follow-up, the disease had not progressed. There were 25 patients who received second-line treatment, with the major reasons being disease stasis or progression.

The response to antifungal treatment was evaluated amongst the patients who were purely treated with antifungal medications. Patients who did not undergo treatment, had received surgical resection of lung lesions, were lost to follow-up, or died of non-cryptococcosis diseases prior to completing treatment were excluded. The median duration of antifungal treatment was 183.0 days (95% CI 179.2–186.8). Amongst the 218 patients, 39 (17.9%) achieved complete response, 136 (62.4%) achieved partial response, 28 (12.8%) had stable disease, and 15 (6.9%) showed progression of disease.

The median duration of follow-up was 823.0 days (95% CI 609.6–1036.4). Regarding overall outcome, 256 patients (79.8%) completed the treatment without recurrence, 4 (1.2%) remained alive with disease recurrence, 8 (2.5%) died of cryptococcosis, and 33 (10.3%) died of non-cryptococcosis disease (two of them had experienced disease recurrence).

### 3.5. Factors Correlated with Radiological Presentations and Extent of Disease

The results of univariate analysis on radiological presentations and the extent of disease are summarized in Table 5. ROC curves were conducted for evaluating the CrAg test of blood in predicting the extent of disease and outcome (Supplementary Figure S2). Accordingly, we chose 1:128 as the cut-off level.

Patients aged ≥65 years were less likely to present with solitary lung nodule or mass and were more likely to have multilobar involvement (both *p* < 0.001). Gender and smoking behavior did not matter in radiological presentations or extent of disease.

Patients having more comorbidities were more likely to have multilobar involvement and extrapulmonary involvement (*p* < 0.001 and 0.004, respectively). Although there were numerically higher rates of cavitation and lower rates of single nodule or mass in patients with multiple comorbidities, both *p* values were not significant.

Patients with high CrAg titer in blood had a significantly higher rate of multilobar and extrapulmonary involvement (both *p* < 0.001) and a significantly lower rate of single nodule or mass presentation (*p* = 0.001). There was no significant association with cavitation.

In multivariate analysis, a CrAg test in blood ≥ 1:128 independently predicted both multilobar involvement (aOR 2.58 (95% CI 1.48–4.47), *p* = 0.001) and extrapulmonary involvement (aOR 12.29 (95% CI 3.89–38.84), *p* < 0.001). Age ≥ 65 years also predicted multilobar involvement (aOR 3.43 (95% CI 1.99–5.92), *p* < 0.001). Comorbidities were associated with significantly more extrapulmonary involvement (aOR 5.67 (95% CI 1.20–26.81), *p* = 0.029), as well as a trend towards more multilobar involvement (aOR 1.66 (95% CI 0.93–2.95), *p* = 0.084).

Herein, we observed that patients who did not present with a solitary nodule or mass, along with those experiencing cavitary lesions, multilobar involvement, and extrapulmonary involvement, were more likely to be clinically symptomatic (*p* = 0.003, 0.006, 0.007, and 0.009, respectively).

### 3.6. Factors Associated with the Outcome of Treatment

Extrapulmonary cryptococcosis, whether found in the CNS or other sites, is well known to be associated with a poor prognosis [14,15]. In our study, extrapulmonary involvement was an independently prognostic factor in predicting recurrence or death due to cryptococcosis (aOR 15.93 (95% CI 3.47–73.05), *p* < 0.001) and recurrence or death from any cause (aOR 3.63 (95% CI 1.11–11.91), *p* = 0.033) among the overall population. CNS was the most common site of extrapulmonary involvement. However, less than half of patients underwent lumbar puncture, with only four patients (1.2%) experiencing neurological symptoms. Hence, we attempted to identify other risk factors.

After exclusion of patients with extrapulmonary involvement, the results of univariate analysis regarding treatment response and outcomes are summarized in Table 6. Smokers, subjects with multiple comorbidities, and those with a high CrAg titer were less likely to have their lesions controlled by antifungal treatment (*p* = 0.028, 0.008, and 0.005, respectively). There was also a trend towards more disease progression in the elderly (*p* = 0.071). The elderly, patients with more comorbidities, and those with a high CrAg titer were more likely to experience disease recurrence or death from any cause (*p* = 0.008, <0.001, 0.010, respectively). In multivariate analysis, patients with a CrAg test ≥ 1:128 (aOR 0.11 (95% CI 0.02–0.68), *p* = 0.018) and multiple comorbidities (aOR 0.37 (95% CI 0.16–0.84, *p* = 0.018) independently predicted a lower disease control rate. Smoking status did not matter in the response among the multivariate model. Both age ≥ 65 years (aOR 2.68 (95% CI 1.09–6.58), *p* = 0.031) and multiple comorbidities (aOR 2.30 (95% CI 1.51–3.52), *p* < 0.001) were independently associated with recurrence or death from any cause. There was also a trend towards a poor outcome in the CrAg test ≥ 1:128 (aOR 2.26 (95% CI 0.91–5.62), *p* = 0.079). Of note, as we considered the impact of individual comorbidities, patients with diabetes mellitus (OR 1.33 (95% CI 1.10–1.61), *p* = 0.004), malignant solid tumor (OR 1.32 (95% CI 1.14–1.52), *p* < 0.001), autoimmune disease (OR 1.23 (95% CI 1.01–1.50), *p* = 0.037), and possible chronic kidney disease (OR 1.47 (95% CI 0.99–2.19), *p* = 0.055) carried a higher risk of recurrence or death from any cause than those without comorbidity.

**Figure 1:**
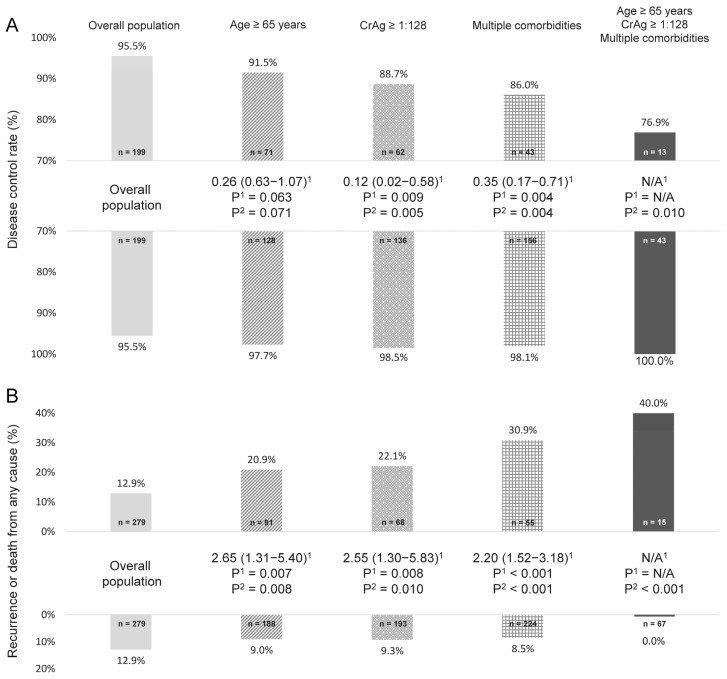
Age, cryptococcal antigen (CrAg) test of blood, comorbidities, and their association with the outcome of isolated pulmonary cryptococcosis: disease control rate (**A**) and recurrence or death from any cause (**B**). ^1^ By logistic regression analysis and data were presented as odds ratio (95% confidence interval). ^2^ By Fisher’s exact test. Upper bars denoted “with” and lower denoted “without” the preset condition(s) for analysis. N/A, not applicable.

When considering these risk factors together, a worse prognosis can be observed in the elderly, as well as those with a high CrAg titer or multiple comorbidities (Figure 1). Of note, patients simultaneously at an age ≥ 65 years, having a CrAg test ≥ 1:128, and with multiple comorbidities experienced the worst outcome (disease control in only 76.9% of patients and recurrence or death from any cause in 40.0%, respectively). By contrast, patients who did not possess any of these risk factors had an excellent treatment response and outcome.

## 4. Discussion

Since the majority of cryptococcal infections develop in HIV-infected patients, the epidemiology, clinical presentations, treatments, and outcomes are clearer and more definite for the HIV-positive population [4,6,16,17]. Currently, most of the information regarding non-HIV-related cryptococcal diseases has come from retrospective studies or case series. Data regarding pulmonary cryptococcosis are even rarer, as cryptococcosis also involves the CNS more than the lungs in non-HIV patients [18,19]. The evidence and consensus guiding the diagnosis and management are not yet well established. Compared with prior reports, our study cohort was larger, and we described the characteristics of non-HIV pulmonary cryptococcosis in detail. Moreover, we explored the relationship between patients’ clinical presentations, radiological findings, and underlying comorbidities, while also identifying patients with a poor prognosis. In addition to extrapulmonary involvement, we suggested that the elderly, those with high CrAg titer in blood, and patients with multiple comorbidities were associated with a worse outcome.

Similarly, we found that cough, fever, chest pain, and shortness of breath were the most common symptoms, and that a significant portion of patients were asymptomatic [9,18,20,21]. Chang et al. and Yu et al. both reported that immunocompetent patients with pulmonary cryptococcosis were more likely to be asymptomatic when compared with immunocompromised hosts [10,21]. We further recognized that patients experiencing comorbidity, particularly those with multiple comorbidities, had a significantly greater extent of disease, including more multilobar and extrapulmonary involvement, which could explain these patients being more symptomatic. Of note, approximately two-thirds of non-HIV patients had at least one comorbidity and were not considered as being truly physically healthy.

Previous studies have suggested that the most common radiological pattern of pulmonary cryptococcosis was multiple nodules or masses [8,10,22], which also accounted for more than half of the computed tomography findings in our cohort. However, less than 40% of patients had conditions involving multiple lobes and less than 30% had involvement in both lungs. These observations imply that the distribution of these lung nodules was usually clustered rather than random (Supplementary Figure S1A), which would differentiate them from that seen in hematogenously spreading metastatic nodules [23]. Moreover, the margin of these nodules was not as well defined as that seen in metastatic pulmonary nodules [11,24], and could sometimes be fluffy.

Although non-HIV patients with pulmonary cryptococcosis generally have a good prognosis, it is still necessary to remain cautious for any possibility of extrapulmonary infection. Of our patients, 6.9% had extrapulmonary involvement, which was much lower than that seen in patients with HIV infection [25,26]. However, once the infection spreads away from the lungs, the prognosis becomes much worse [14]. Our data indicate that extrapulmonary involvement is an independently poor prognostic factor. Current treatment guidelines encourage lumbar puncture for non-immunocompromised patients with pulmonary cryptococcosis to exclude asymptomatic meningitis, but it has not been advised to be mandatory [4,27]. It is possible that some CNS infections would be overlooked. In addition to documented extrapulmonary infection, we also have to recognize risk factors which can predict a poor prognosis for isolated pulmonary cryptococcosis.

Our data suggest that most patients can achieve disease control through antifungal treatment, which is similar to prior reports [9,28]. However, there are limited studies evaluating the outcome predictors among patients with isolated pulmonary cryptococcosis [22]. In the present study, we suggested that age, blood CrAg test, and comorbidities could act as risk factors towards predicting not only a higher extent of disease but also a worse outcome of treatment. Of note, patients simultaneously experiencing old age, high blood CrAg titer, and multiple comorbidities had the lowest disease control rate and highest rate of recurrence or death. The radiological patterns did not influence the response and outcome.

There are studies suggesting smoking as a risk factor for invasive fungal infection [29,30]. Although smokers had a lower disease control rate, it no longer played a significant role in the multivariate model. Furthermore, smoking status neither correlated with the extent of disease nor its outcome. The role of smoking in the acquisition and prognosis of cryptococcosis remains uncertain. In our study, we selected disease control as one of the outcome parameters because patients with stable disease also achieved a minor response (0–25%), and their recurrence or death rate did not differ from that of patients with an objective response. We did not evaluate the efficacy of specific antifungal mediations because we had observed a significant correlation between the severity of disease and regimen choice. Although fluconazole remains the main treatment option in clinical practice [4,31], prospective studies are still required in order to provide more evidence surrounding recommendations for the most appropriate therapy.

Arguments as to whether isolated pulmonary cryptococcosis needs to be treated, and whether the lung lesions are true infections or a colonization, exist [32]. Approximately 95% of our patients had available CrAg results and more than 80% of them were positive, implying there was deep tissue invasion according to the IDSA guideline [4]. In patients with a negative test, 47.2% were symptomatic and many of the remaining patients experienced comorbidity; therefore, clinically, we treated them as having true infections. Prospective studies and updated guidelines are still required in order to better define the indication of treatment.

Herein, we suggested that patients experiencing comorbidity, particularly those with multiple comorbidities, had a worse outcome. When we looked at the impact of individual comorbidities, diabetes mellitus, malignant solid tumor, autoimmune disease, and possible chronic kidney disease served as poor prognostic factors. Previous studies suggested that uncontrolled diabetes mellitus was associated with a worse outcome of cryptococcosis [33,34]. Data regarding other comorbidities were limited. Because a significant portion of patients actually had more than one comorbidity, the comparison between each comorbidity may require further research.

## 5. Conclusions

Non-HIV pulmonary cryptococcosis patients, particularly those without extrapulmonary involvement, generally had favorable outcomes. Herein, we demonstrated the correlation between patients’ clinical presentations, radiological findings, and underlying comorbidities. In addition to extrapulmonary involvement, old age, high CrAg titer in blood, and multiple comorbidities also predicted a higher extent of disease and a worse outcome.

## Figures and Tables

**Table 1:** Demographic data and patients’ characteristics.

Characteristics	*N* = 321
**Age (years)**	median (range)
	59 (20–87)
**Gender**	*n* (%)
Female	138 (43.0)
Male	183 (57.0)
**Smoking status**	*n* (%)
Non-smokers	234 (72.9)
Smokers	68 (21.2)
Unknown	19 (5.9)
**Comorbidities**	*n* (%)
No	117 (36.4)
Yes	204 (63.6)
Single	137 (42.7)
Multiple	67 (20.9)
Diabetes mellitus	88 (27.4)
Malignant solid tumor ^$^	63 (19.6)
Autoimmune disease ^&^	50 (15.6)
Chronic kidney disease *	27 (8.4)
Hematological disorder ^#^	17 (5.3)
Chronic lung disease	15 (4.7)
Chronic liver disease	10 (3.2)
Cardiovascular disease	8 (2.5)
Organ transplantation	3 (0.9)
Alcoholism	2 (0.6)
Common variable immune deficiency	2 (0.6)

* Includes 4 patients under hemodialysis. ^#^ Includes 13 and 4 patients with malignant and non-malignant hematological disorders, respectively. ^$^ The most common are lung cancer, colorectal cancer, and breast cancer. ^&^ The most common are rheumatoid arthritis, systemic lupus erythematosus, and Sjogren’s syndrome.

**Table 2:** Clinical and radiological presentations of pulmonary cryptococcosis.

Clinical Symptoms	*N* = 321, *n* (%)
No	111 (34.6)
Yes	210 (65.4)
Cough	129 (40.2)
Fever	42 (13.1)
Shortness of breath	23 (7.2)
Chest pain	21 (6.5)
Hemoptysis	10 (3.1)
Dizziness/headache	10 (3.1)
Decreased appetite/weight loss	8 (2.5)
Fatigue	7 (2.2)
Chillness	6 (1.9)
Altered consciousness/neurological deficit	4 (1.2)
**Extent of disease**	***N* = 321, *n* (%)**
Extrapulmonary involvement	
No	299 (93.1)
Yes	22 (6.9)
Central nervous system (CNS)	18 (5.6)
Fungemia	8 (2.5)
Others *	4 (1.2)
**Findings on chest computed tomography**	***N* = 305, *n* (%)**
Extent of pulmonary cryptococcosis	
Single lobe	185 (60.7)
Multiple lobes ^#^	120 (39.3) ^#^
Radiological presentations	
Multiple nodules/masses with or without cavitation,^$^	161 (52.8)
Consolidation	52 (17.0)
Solitary nodule/mass with or without cavitation ^$^	48 (15.7)
Ground glass opacity	3 (1.0)
Mixed pattern ^$,&^	41 (13.4)
Associated findings	
Pleural effusion	11 (3.6)
Lymphadenopathy	5 (1.6)

* Includes 1 with urinary tract infection, 1 with paraspinal abscess, and 2 with neck lymph node infection. ^#^ Includes 88 patients with involvement of both lungs. ^$^ Among patients with nodule(s) or mass(es), a total of 37 patients with cavitation. ^&^ Most common are consolidation mixed with nodule(s)/mass(es) (*n* = 29).

**Table 3:** The patterns and methods of pulmonary cryptococcosis diagnosis.

CrAg Test of Blood, *n* (%)	*N* = 321
Not performed	18 (5.6)
Yes	303 (94.4)
Positive *	250 (82.5)
Negative	53 (17.5)
**Culture and identification of species, *n* (%)**	***N* = 321**
Not performed	167 (52.0)
Yes	154 (48.0)
Cerebrospinal fluid (CSF), total/positive ^$^	110/13
Tissue, total/positive	62/17
Bronchoalveolar lavage, total/positive	21/13
Sputum, total/positive	19/4
Blood, total/positive	13/8
Pleural effusion, total/positive	2/0
Urine, total/positive	1/1
Identifiable culture results (*n* = 47)	
*Cryptococcus neoformans*	46 (97.9)
*Cryptococcus gattii*	1 (2.1)
**Biopsy and pathology, *n* (%)**	***N* = 321**
Not performed	186 (57.9)
Yes	135 (42.1)
Surgical biopsy, total/positive	64/64
Computed-tomography-guided biopsy, total/positive	28/24
Bronchoscopy, total/positive ^#^	23/7
Ultrasound-guided biopsy, total/positive	20/12
**Lumbar puncture, *n* (%)**	***N* = 321**
Not performed	211 (65.7)
Yes	110 (34.3)
CrAg test of CSF negative	92 (83.6)
CrAg test of CSF positive ^$^	18 (16.4)

CrAg, cryptococcal antigen. * Denotes the percentage of positivity among patients with serology tests (median 1:32 (range 1:2 to 1:8192)). ^#^ Includes bronchoscopic biopsy, brush, and/or lavage. ^$^ All 18 patients with a positive CSF CrAg test also had positive results in the blood; all 13 patients with positive CSF culture were also positive for the CSF CrAg test.

**Table 4:** The patterns of treatment and outcome of pulmonary cryptococcosis.

First-Line Treatment, *n* (%)	*N* = 321
No treatment	5 (1.6)
Surgical resection only	34 (10.6)
Antifungal treatment	282 (87.9)
Fluconazole *	251 (89.0)
Amphotericin-B-containing regimen ^#^	31 (11.0)
**Second-line treatment, *n* (%)**	***N*** **= 25**
Reasons for second-line treatment	
Disease stasis or progression	21 (84.0)
Intolerance of first-line treatment	2 (8.0)
Exclusion of malignancy	2 (8.0)
Types of treatment	
Surgical resection	13 (52.0)
Amphotericin-B-containing regimen	7 (28.0)
Add-on flucytosine	3 (12.0)
Azoles other than fluconazole	2 (8.0)
**Outcome of first-line antifungal treatment ^$^**	***N*** **= 218**
Complete response	39 (17.9)
Part response	136 (62.4)
Stable disease	28 (12.8)
Disease progression	15 (6.9)
**Overall outcome**	***N*** **= 321**
Complete treatment without recurrence	256 (79.8)
Alive with disease recurrence	4 (1.2)
Death	41 (12.8)
Cryptococcosis related	8 (2.5)
Not cryptococcosis related ^&^	33 (10.3)
Incomplete treatment and loss of follow-up	20 (6.2)

* Includes 28 patients who underwent surgical biopsy or resection before antifungal treatment. ^#^ Four patients did not undergo fluconazole maintenance because of early death; all of them had underlying autoimmune disease and 3 of them had fungemia. ^$^ Excludes patients without treatment, with surgical resection, loss of follow-up, and not-cryptococcosis-related death before complete treatment. ^&^ Includes 2 patients with recurrent cryptococcosis.

**Table 5:** Univariate analysis of radiological presentations and the extent of disease.

Factors		*N* ^#^	Cavitation	*p* Value *	Single Nodule or Mass	*p* Value *	Multilobar Involvement	*p* Valve *	*N* ^$^	Extrapulmonary Involvement	*p* Value *
Age	<65 years	198	25 (12.6)	0.855	42 (21.2)	<0.001	54 (27.3)	<0.001	208	12 95.8)	0.356
	≥65 years	107	12 (11.2)		6 (5.6)		66 (61.7)		113	10 (8.8)	
Gender	Female	129	15 (11.6)	0.861	25 (19.4)	0.153	45 (34.9)	0.193	138	9 (6.5)	1.000
	Male	176	22 (12.5)		23 (13.1)		75 (42.1)		183	13 (7.1)	
Smoking status	Non-smokers	223	25 (11.2)	0.664	37 (16.6)	0.702	86 (38.6)	0.475	234	16 (6.8)	0.793
	Smokers	66	9 (13.6)		9 (13.6)		29 (43.9)		68	5 (7.4)	
Comorbidities	No	113	10 (8.8)	0.383	22 (19.5)	0.177	29 (25.7)	<0.001	117	2 (1.7)	0.004
	Single	126	18 (14.3)		20 (15.9)		55 (43.7)		137	11 (8.0)	
	Multiple	66	9 (13.6)		6 (9.1)		36 (54.5)		67	9 (13.4)	
Symptoms	No	110	6 (5.5)	0.006	27 (24.5)	0.003	32 (29.1)	0.007	111	2 (1.8)	0.009
	Yes	195	31 (15.6)		21 (10.8)		88 (45.1)		210	20 (9.5)	
Blood CrAg test	<1:128	203	21 (10.3)	0.115	35 (17.2)	0.001	69 (34.0)	<0.001	215	5 (2.3)	< 0.001
	≥1:128	84	15 (17.9)		3 (3.6)		50 (59.5)		88	17 (19.3)	

CrAg, cryptococcal antigen. * By Fisher’s exact test. ^#^ Analysis of radiological presentations. ^$^ Analysis of extrapulmonary involvement.

**Table 6:** Univariate analysis of the treatment response and overall outcomes among patients with isolated pulmonary cryptococcosis.

Factors		*N* ^#^	Disease Control Rate	*p* Value *	*N* ^$^	Recurrence or Death from any Cause	*p* Value *
Age	<65 years	128	125 (97.7)	0.071	188	17 (9.0)	0.008
	≥65 years	71	65 (91.5)		91	19 (20.9)	
Gender	Female	84	82 (97.6)	0.307	117	11 (9.4)	0.152
	Male	115	108 (93.9)		162	25 (15.4)	
Smoking status	Non-smokers	145	141 (97.2)	0.028	207	24 (11.6)	0.269
	Smokers	42	37 (88.1)		58	10 (17.2)	
Comorbidities	No	72	71 (98.6)	0.008	111	4 (3.6)	<0.001
	Single	84	82 (97.6)		113	15 (13.3)	
	Multiple	43	37 (86.0)		55	17 (30.9)	
Blood CrAg test	<1:128	136	134 (98.5)	0.005	193	18 (9.3)	<0.010
	≥1:128	62	55 (88.7)		68	15 (22.1)	
Lung involvement	Single lobe	113	110 (97.3)	0.179	170	19 (11.2)	0.263
	Multilobar	86	80 (93.0)		99	16 (16.2)	
Cavitation	No	175	168 (96.0)	0.297	238	30 (12.6)	0.572
	Yes	24	22 (91.7)		31	5 (16.1)	
Single nodule or mass	No	185	176 (95.1)	1.000	224	30 (13.4)	0.811
	Yes	14	14 (100.0)		42	5 (11.1)	

CrAg, cryptococcal antigen.* By Fisher’s exact test. ^#^ Analysis of disease control; excludes patients without treatment, with surgical resection, loss of follow-up, and not-cryptococcosis-related death before complete treatment. ^$^ Analysis of recurrence of death; excludes patients with loss of follow-up.

## Data Availability

The data presented in this study are available on request from the corresponding author. The data are not publicly available due to restriction of corresponding IRB.

## Supplementary Materials

The following are available online at https://www.mdpi.com/article/10.3390/jof7080657/s1, Figure S1: Common computed tomography findings of pulmonary cryptococcosis. Figure S2: Receiver-operating characteristic (ROC) curve for evaluating Latex agglutination test of blood in predicting the extent of disease and outcome
