# Supplementary material for: Comprehensive Analysis and Risk Identification of Pulmonary Cryptococcosis in Non-HIV Patients
Source: J Fungi (Basel). 2021 Aug 13;7(8):657. doi: 10.3390/jof7080657 (PMC8399630; doi:10.3390/jof7080657)
Supplement: Supplementary file 1 [file jof-07-00657-s001.zip › jof-1323722-supplementary.pdf]

Journal of *Fungi*

Supplementary Supplementary

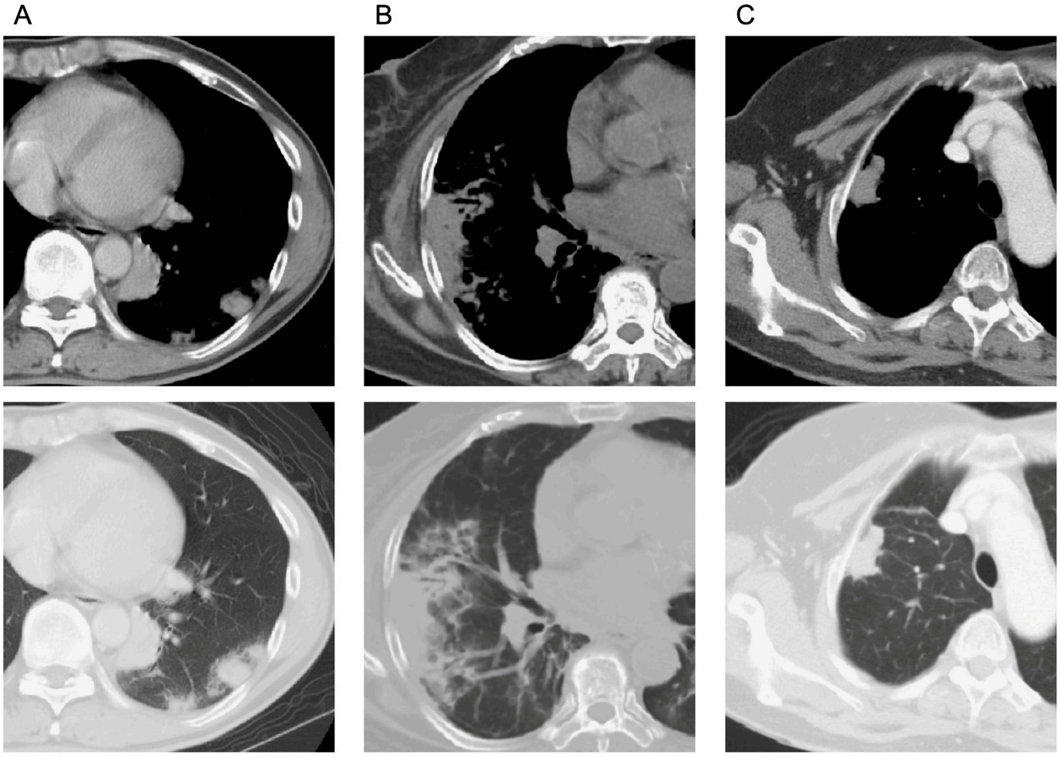

Figure S1. Common computed tomography findings of pulmonary cryptococcosis: multiple nodules/masses with or without cavitation (A), consolidation (B), and solitary nodule/mass with or without cavitation (C).

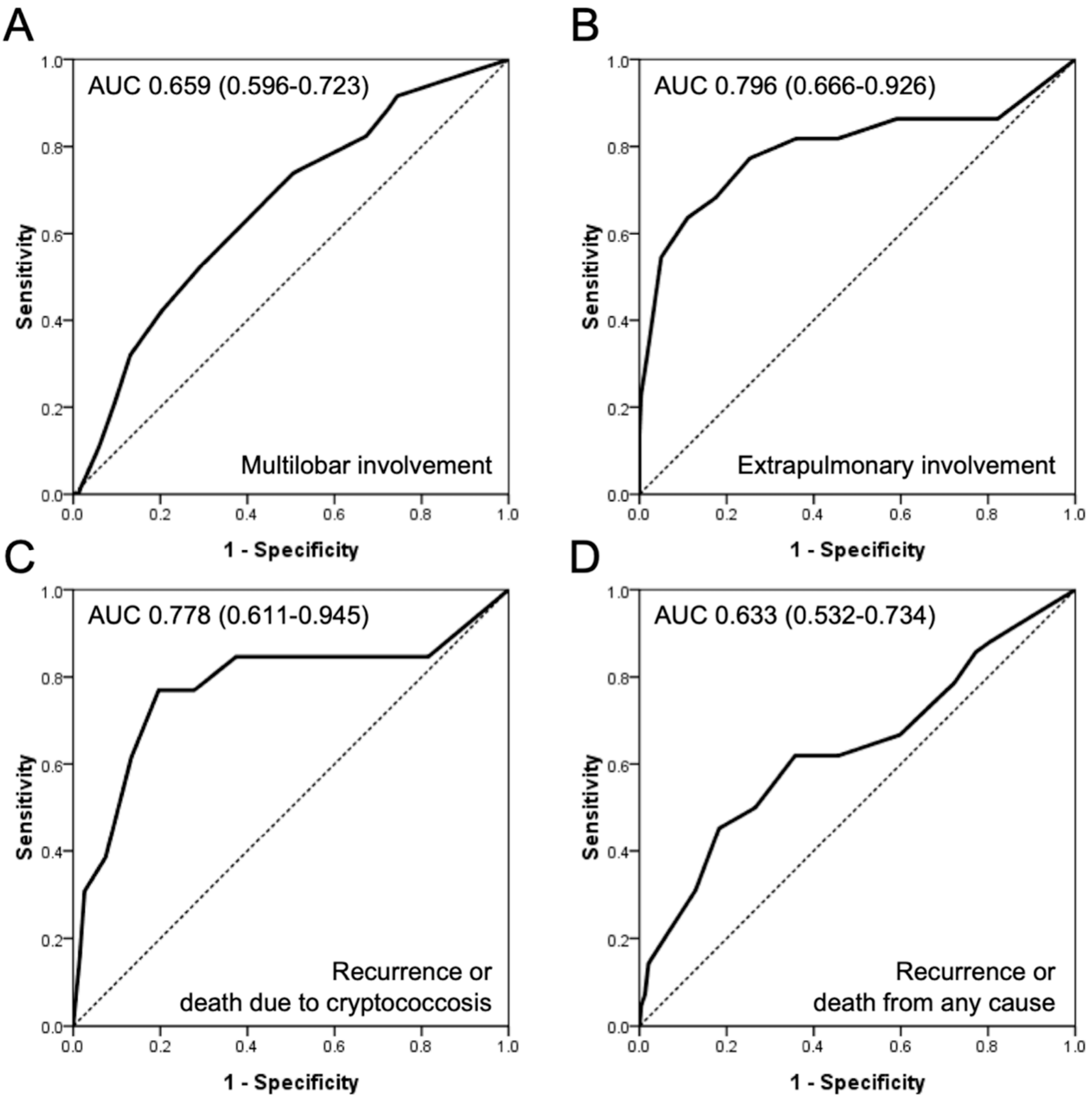

Figure S2. Receiver-operating characteristic (ROC) curve for evaluating Latex agglutination test of blood in predicting the extent of disease and outcome.
